# Clinical features and predictors of outcome in patients with acute myocardial infarction complicated by out-of-hospital cardiac arrest

**DOI:** 10.1186/s12872-022-02628-3

**Published:** 2022-04-19

**Authors:** Taketo Sonoda, Hideki Wada, Manabu Ogita, Daigo Takahashi, Ryota Nishio, Kentaro Yasuda, Mitsuhiro Takeuchi, Shoichiro Yatsu, Jun Shitara, Shuta Tsuboi, Tomotaka Dohi, Satoru Suwa, Katsumi Miyauchi, Tohru Minamino

**Affiliations:** 1 Department of Cardiovascular Medicine, Juntendo University Shizuoka Hospital, 1129 Nagaoka, Izunokuni, Shizuoka 410-2295 Japan; 2 Department of Cardiovascular Medicine and Biology, Juntendo University Graduate School of Medicine, Tokyo, Japan

**Keywords:** Acute myocardial infarction, Cardiac arrest, Percutaneous coronary intervention

## Abstract

**Background:**

Although short-term mortality of acute myocardial infarction (AMI) has decreased dramatically in the past few decades, sudden cardiac arrest remains a serious complication. The aim of the study was to assess the clinical characteristics and predictors of prognosis in AMI patients who experienced out-of-hospital cardiac arrest (OHCA).

**Methods:**

We retrospectively registered consecutive AMI patients who were treated with emergency percutaneous coronary intervention (PCI) between 2004 and 2017. Clinical characteristics and outcomes were compared between patients with OHCA and those without OHCA.

**Results:**

Among 2101 AMI patients, 95 (4.7%) presented with OHCA. Younger age (odds ratio [OR]: 0.95; 95% confidence interval [CI]: 0.93–0.97; *p* < 0.0001), absence of diabetes mellitus (OR, 0.51; 95% CI, 0.30–0.85; *p* = 0.01) or dyslipidemia (OR, 0.56; 95% CI, 0.36–0.88; *p* = 0.01), left main trunk (LMT) or left anterior descending artery (LAD) as the culprit lesion (OR, 3.26; 95% CI, 1.99–5.33; *p* < 0.0001), and renal deficiency (OR, 3.64; 95% CI, 2.27–5.84; *p* < 0.0001) were significantly associated with incidence of OHCA. Thirty-day mortality was 32.6% in patients with OHCA and 4.5% in those without OHCA. Multivariate logistic analysis revealed LMT or LAD as the culprit lesion (OR, 12.18; 95% CI, 2.27–65.41; *p* = 0.004), glucose level (OR, 1.01; 95% CI, 1.00–1.01; *p* = 0.01), and renal deficiency (OR, 3.35; 95% CI, 1.07–10.53; *p* = 0.04) as independent predictors of 30-day mortality among AMI patients with OHCA.

**Conclusions:**

In patients with AMI who underwent emergency PCI, 30-day mortality was six times greater in those having presented initially with OHCA compared with those without OHCA. Younger age, absence of diabetes mellitus or dyslipidemia, LMT or LAD as the culprit lesion, and renal deficiency were independent predictors of OHCA. OHCA patient with higher blood glucose level on admission, LMT or LAD as the culprit lesion, or renal deficiency showed worse clinical outcomes.

## Background

Acute myocardial infarction (AMI) is associated with high mortality and remains a major public health problem despite the development of reperfusion therapies such as coronary catheter interventions and optimal medications based on universal guidelines [1, 2]. In the 1970s–80s, ST-elevation myocardial infarction (STEMI) with shock was a fatal condition and its survival rate was only 20% [3, 4]. Even in present times, mortality in patients with STEMI approaches 50% and outcomes have shown no improvement over the past two decades [5, 6]. In particular, AMI complicated by out-of-hospital cardiac arrest (OHCA) remains a serious life-threating condition. It has been associated with equal mortality to patients in cardiogenic shock, and is reported to have ten times the mortality of patients without complicating cardiac arrest (CA) [7–9]. In addition, it has been reported that approximately 70% of CA patients have coronary artery disease (CAD), nearly 50% of which is associated with AMI as acute occlusion of the coronary artery vessels [10–12]. Investigating the risk stratification of AMI patients who present with OHCA might help improve the mortality rate of AMI, and potentially that of OHCA. However, there are few data for AMI complicated with OHCA, especially in Asian populations. Therefore, the aim of the present study was to investigate the clinical characteristics and predictors of prognosis of AMI patients who present with OHCA.

## Methods

### Patients and data collection

We performed a single-center observational study of consecutive patients who underwent emergency percutaneous coronary intervention (PCI) for AMI at our institution between 2004 and 2017. Therefore, we excluded patients in whom AMI was not suspected and the AMI culprit lesion was not identified by coronary angiography (CAG). The diagnosis of AMI was based on the criteria of the European Society of Cardiology/American College of Cardiology Foundation/American Heart Association/World Heart Federation Task Force for the Universal Definition of Myocardial Infarction [13]. Only type 1 myocardial infarction (MI; spontaneous MI related to ischemia due to a primary coronary event) was included in this study. STEMI was diagnosed when new ST-elevation at the J point was present in at least two contiguous leads. Patients with clinical suspicion of ongoing myocardial ischemia and left bundle branch block were considered equivalent to STEMI patients and were managed in a similar way [14]. Patients without ST-segment elevation at presentation were designated as non-ST-elevation myocardial infarction (NSTEMI).

Demographic data and information regarding coronary risk factors, medications, revascularization procedure-related factors, and comorbidities were collected retrospectively from the patient medical record database at Juntendo University Shizuoka Hospital. Blood samples were collected before emergency PCI, and blood pressure (BP) was measured on admission. Patients with BP > 140/90 mmHg or those receiving antihypertensive drugs were regarded as hypertensive [15]. Dyslipidemia was defined as low-density lipoprotein cholesterol (LDL-C) ≥ 140 mg/dL, high-density lipoprotein cholesterol (HDL-C) ≤ 40 mg/dL, triglycerides ≥ 150 mg/dL, or current treatment with statins and/or lipid-lowering agents [16]. Diabetes mellitus was defined as either hemoglobin A1c ≥ 6.5% or medication with insulin or oral hypoglycemic drugs [17]. Renal deficiency was defined as an estimated glomerular filtration rate (eGFR) at admission of < 60 mL/min/1.73 m^2^, calculated using the Modification of Diet in the Renal Disease equation modified with a Japanese coefficient using baseline serum creatinine [18]. A current smoker was defined as a person who was a smoker at the time of admission or who had quit smoking within 1 year prior to admission.

This study was approved by the Institutional Review Board of our hospital and was performed in accordance with the Declaration of Helsinki. All participants provided written informed consent.

### Primary endpoint

The primary endpoint was 30-day all-cause mortality. Mortality data were collected from the medical records of patients who died or who were treated at our institution. These details and the causes of death were obtained from other hospitals in the case of patients who had been transferred.

### Statistical analysis

Quantitative data are expressed as the mean ± standard deviation or the median and interquartile range, and categorical variables are expressed as number and percentage. Continuous variables were compared using an unpaired t-test or the Mann–Whitney U-test. Categorical variables were compared using the chi-square test or Fisher’s exact probability test. Logistic regression analysis was performed to clarify the factors associated with the incidence of OHCA. Related variables in the univariate analysis were used as variables in the multivariable logistic regression analysis, which was used to calculate the odds ratio (OR) and 95% confidence interval (CI). Survival curves were estimated using the Kaplan–Meier method and the log-rank test was used to detect statistically significant difference. Among patients with OHCA, multivariate logistic regression analysis was performed to identify the predictors of 30-day mortality. Variables that were included in the multivariate model were selected by univariate logistic regression analysis (*p* < 0.05). A *p* value < 0.05 was taken to indicate statistical significance. All statistical analysis was performed using JMP 14.0 (SAS Institute Inc., Cary, NC, USA).

## Results

### Baseline characteristics

**Fig. 1:**
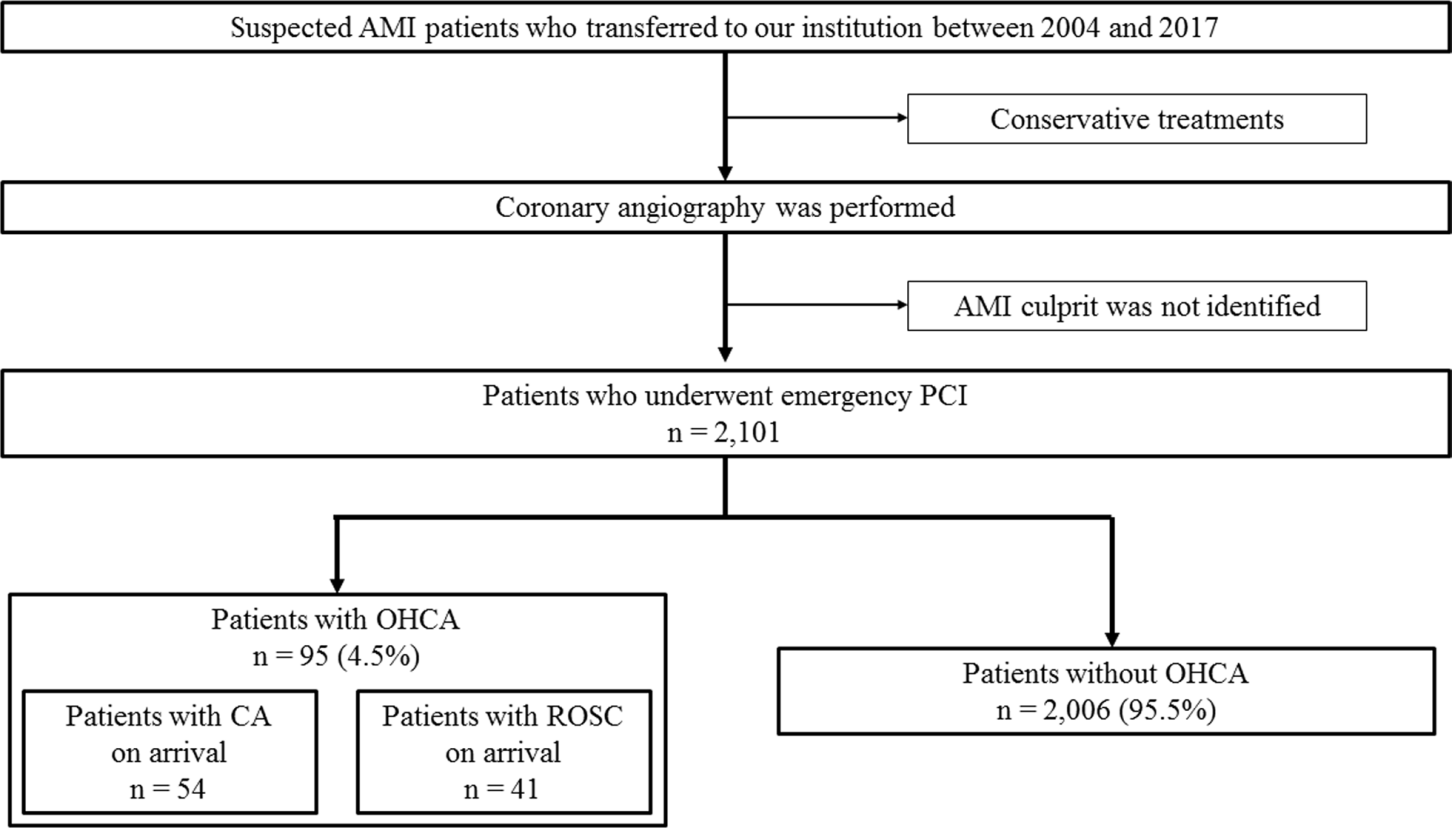
Flow chart of the study. The subjects were 2101 consecutive AMI patients who underwent emergency PCI between 2004 and 2017. When divided into two groups according to incidence of OHCA, 95/2101 (4.5%) patients presented with OHCA. *AMI* acute myocardial infarction, *OHCA* out-of-hospital cardiac arrest, *PCI* percutaneous coronary intervention, *ROSC* return of spontaneous circulation

The study population included 2101 consecutive patients with AMI who underwent emergency PCI between 2004 and 2017, of whom 95 (4.5%) presented with OHCA (Fig. 1). Among these, 54 patients (56.8%) were resuscitated successfully before admission. The baseline and procedural characteristics of these patients are shown in Table 1. Compared with patients without OHCA (non-OHCA group), patients with OHCA (OHCA group) were more likely to be younger, male, and have renal deficiency, but less likely to have a history of traditional coronary risk factors such as diabetes mellitus or dyslipidemia. Blood glucose and white blood cell counts were significantly higher in the OHCA group, and brain natriuretic peptide levels were significantly lower in these patients. The culprit lesion was more likely to be in the LMT or LAD in the OHCA group. There was no significant difference in the rate of multivessel CAD between the groups. The use of mechanical support such as intra-aortic balloon pumping or percutaneous cardiopulmonary support was more frequent in the OHCA group than the non-OHCA group.

**Table 1:** Baseline clinical characteristics

	Overall (n = 2101)	OHCA (n = 95)	Non-OHCA (n = 2006)	*p* value
Age, y	68.5 ± 12.1	63.2 ± 13.8	68.7 ± 12.0	< 0.0001
Male, n (%)	1588 (75.6)	83 (87.4)	1505 (75.0)	0.006
Hypertension, n (%)	1426 (68.0)	60 (64.5)	1366 (68.2)	0.46
Diabetes mellitus, n (%)	784 (37.4)	24 (25.5)	760 (37.9)	0.02
Dyslipidemia, n (%)	1245 (59.3)	45 (47.9)	1200 (59.9)	0.02
Current smoker, n (%)	853 (40.7)	43 (46.2)	810 (40.5)	0.27
Family history of CAD, n (%)	369 (18.0)	15 (17.2)	354 (18.0)	0.86
Multivessel CAD, n (%)	1012 (48.2)	45 (47.4)	967 (48.2)	0.87
LMT or LAD as IRA, n (%)	1044 (49.7)	72 (75.8)	972 (48.5)	< 0.0001
Initial TIMI flow ≥ 2, n (%)	455 (21.7)	20 (21.1)	435 (21.7)	0.88
Cardiogenic shock on arrival, n (%)	129 (6.2)	47 (50.0)	82 (4.1)	< 0.0001
IABP use, n (%)	467 (22.3)	60 (63.2)	407 (20.3)	< 0.0001
PCPS use, n (%)	75 (3.6)	33 (34.7)	42 (2.1)	< 0.0001
STEMI, n (%)	1836 (87.4)	74 (77.9)	1762 (87.8)	0.004
Prior PCI, n (%)	175 (8.4)	8 (8.7)	167 (8.3)	0.90
Prior stroke, n (%)	211 (10.1)	6 (6.5)	205 (10.2)	0.25
Body mass index, kg/m^2^	23.8 ± 3.8	24.8 ± 4.6	23.8 ± 3.8	0.02
Arterial fibrillation, n (%)	175 (8.3)	8 (8.4)	167 (8.3)	0.97
TC, mg/dL	186.6 ± 45.4	177.3 ± 51.9	187.1 ± 45.1	0.05
LDL-C, mg/dL	117.8 ± 37.5	110.1 ± 40.3	118.2 ± 37.3	0.05
HDL-C, mg/dL	47.2 ± 13.4	40.1 ± 12.1	47.5 ± 13.4	< 0.0001
Triglyceride, mg/dL	77 [49, 123]	110 [68, 171]	75 [49, 120]	< 0.0001
Glucose, mg/dL	180.1 ± 84.0	259.1 ± 118.2	176.4 ± 80.2	< 0.0001
HbA1c, %	6.3 ± 1.3	6.2 ± 1.1	6.3 ± 1.4	0.32
BNP, ng/dL	93 [28, 267]	46 [15, 116]	96 [29, 272]	0.0001
White blood cells, /mL	10,500 [8400, 13,300]	13,800 [10200, 17,800]	10,400 [8400, 13,100]	< 0.0001
Hemoglobin, g/dL	13.3 ± 2.1	13.5 ± 2.3	13.3 ± 2.1	0.30
eGFR, mL/min/1.73 m^2^	66.4 ± 20.0	58.1 ± 16.7	66.8 ± 20.1	< 0.0001
Renal deficiency, n (%)	741 (36.2)	53 (56.4)	688 (35.2)	< 0.0001
Hemodialysis, n (%)	52 (2.5)	1 (1.1)	51 (2.5)	0.37

*BNP* brain natriuretic peptide, *CAD* coronary artery disease, *eGFR* estimated glomerular filtration rate, *HDL-C* high-density lipoprotein cholesterol, *IABP* intra-aortic balloon pumping, *IRA* infarct related artery, *LAD* left anterior descending, *LDL-C* low-density lipoprotein cholesterol, *LMT* left main trunk, *OHCA* out of hospital cardiac arrest, *PCI* percutaneous coronary intervention, *PCPS* percutaneous cardio pulmonary support, *STEMI* ST-elevation myocardial infarction, *TC* total cholesterol, *TIMI* thrombolysis in myocardial infarction

### Independent factors of OHCA among AMI patients

Table 2 lists the results of univariate and multivariate logistic regression analysis. Age, sex, body mass index (BMI), diabetes mellitus, dyslipidemia, and left main trunk (LMT) or left anterior descending artery (LAD) as the culprit lesion of AMI were selected by univariate logistic regression analysis (*p* < 0.05) and entered into the multivariate model. Multivariate regression analysis revealed younger age (OR, 0.95; 95% CI, 0.93–0.97; *p* < 0.0001), absence of diabetes mellitus (OR, 0.51; 95% CI, 0.30–0.85; *p* = 0.01) and dyslipidemia (OR, 0.56; 95% CI, 0.36–0.88; *p* = 0.01), LMT or LAD as the culprit lesion (OR, 3.26; 95% CI, 1.99–5.33; *p* < 0.0001), and renal deficiency (OR, 3.64; 95% CI, 2.27–5.84; *p* < 0.0001) as factors significantly associated with incidence of OHCA.

**Table 2:** Logistic regression analysis for incidence of OHCA among AMI patients

	Univariate			Multivariate		
	Odds ratio	95% CI	*p* value	Odds ratio	95% CI	*p* value
Age	0.96	0.95–0.98	< 0.0001	0.95	0.93–0.97	< 0.0001
Male	2.30	1.25–4.25	0.008	1.24	0.65–2.39	0.51
Body mass index	1.06	1.01–1.11	0.02	1.03	0.97–1.09	0.39
Current smoker	1.27	0.83–1.92	0.27			
Prior PCI	1.05	0.50–2.20	0.90			
Prior stroke	0.61	0.26–1.42	0.25			
Initial TIMI flow ≥ 2	1.60	0.42–6.26	0.50			
Multivessel CAD	0.97	0.64–1.46	0.87			
LMT or LAD as IRA	3.33	2.07–5.37	< 0.0001	3.26	1.99–5.33	< 0.0001
Hb	1.05	0.95–1.17	0.30			
Hypertension	0.85	0.55–1.31	0.46			
Diabetes mellitus	0.56	0.35–0.90	0.02	0.51	0.30–0.85	0.01
Dyslipidemia	0.62	0.41–0.93	0.02	0.56	0.36–0.88	0.01
Renal deficiency	2.38	1.57–3.61	< 0.0001	3.64	2.27–5.84	< 0.0001

*AMI* acute myocardial infarction, *CAD* coronary artery disease, *CI* confidence interval, *IRA* infarct related artery, *LAD* left anterior descending, *LMT* left main trunk, *OHCA* out of hospital cardiac arrest, *PCI* percutaneous coronary intervention, *TIMI* thrombolysis in myocardial infarction

### Clinical outcomes and predictors of 30-mortality among OHCA patients

**Fig. 2:**
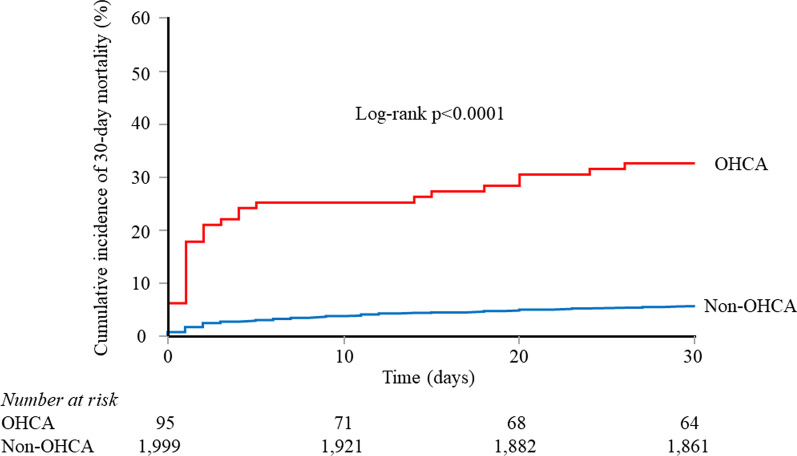
Kaplan–Meier curves for all-cause death. Kaplan–Meier analysis confirms the 30-day mortality was significantly higher in the OHCA group than the non-OHCA group (log-rank *p* < 0.0001). *OHCA* out-of-hospital cardiac arrest

Among the total AMI population, 147/2101 patients (7.0%) died within 30 days of admission. The 30-day mortality was 32.6% in the OHCA group and 5.8% in the non-OHCA group. Kaplan–Meier analysis confirmed that 30-day death was significantly higher in the OHCA group than the non-OHCA group (log-rank *p* < 0.0001, Fig. 2).

Logistic regression analysis of the OHCA group to identify predictors of 30-day mortality revealed renal deficiency, hemoglobin level, glucose level, LMT or LAD as the culprit lesion, and multivessel coronary artery disease as factors for inclusion in the multivariate model. Multivariate logistic analysis indicated renal deficiency (OR, 3.35; 95% CI 1.07–10.53; *p* = 0.04), LMT or LAD as the culprit lesion (OR, 12.18; 95% CI 2.27–65.41; *p* = 0.004), and glucose level on admission (OR, 1.01; 95% CI 1.00–1.01; *p* = 0.01) as independent predictors of 30-day mortality (Table 3).

**Table 3:** Logistic regression analysis for 30-day mortality among AMI patients with OHCA

	Univariate			Multivariate		
	Odds ratio	95% CI	*p* value	Odds ratio	95% CI	*p* value
Age	0.99	0.96–1.03	0.68			
Male	2.69	0.55–13.09	0.22			
ST-elevation MI	0.44	0.16–1.18	0.10			
Body mass index	1.02	0.93–1.13	0.62			
Prior PCI	1.34	0.30–6.02	0.70			
Prior stroke	1.09	0.19–6.33	0.92			
Multivessel CAD	2.84	1.16–6.91	0.02	2.42	0.83–7.04	0.10
LMT or LAD as IRA	7.09	1.54–32.54	0.01	12.18	2.27–65.41	0.004
Hb	0.80	0.66–0.97	0.02	0.83	0.67–1.04	0.10
Glucose	1.01	1.00–1.01	0.002	1.01	1.00–1.01	0.01
Renal deficiency	4.02	1.51–10.68	0.005	3.35	1.07–10.53	0.04

*AMI* acute myocardial infarction, *CAD* coronary artery disease, *CI* confidence interval, *IRA* infarct related artery, *LAD* left anterior descending, *LMT* left main trunk, *MI* myocardial infarction, *OHCA* out of hospital cardiac arrest, *PCI* percutaneous coronary intervention

## Discussion

**Fig. 3:**
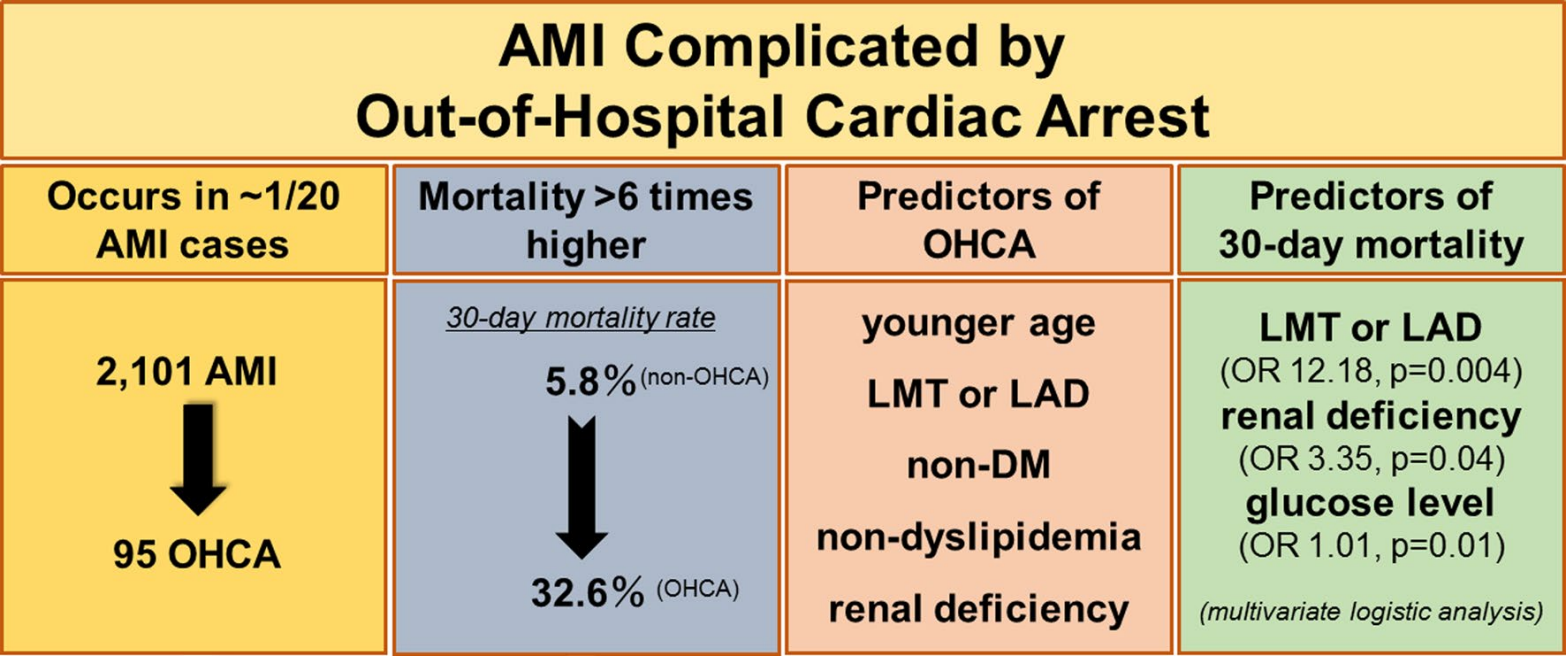
AMI complicated by OHCA: major findings of the present study). Approximately 1 in 20 AMI patients presented with OHCA. Younger age, absence of diabetes mellitus or dyslipidemia, LMT or LAD as the culprit lesion, and renal deficiency were significantly associated with incidence of OHCA. Thirty-day mortality was more than six times higher in the OHCA group than in the non-OHCA group. Renal deficiency, LMT or LAD as the culprit lesion, and higher blood glucose level were independent predictors of 30-day mortality in the OHCA group. *AMI* acute myocardial infarction, *DM* diabetes mellitus, *LAD* left anterior descending artery, *LMT* left main trunk, *OHCA* out-of-hospital cardiac arrest, *PCI* percutaneous coronary intervention

The major findings of the present study were as follows: (1) 4–5% of AMI patients presented with OHCA; (2) younger age, absence of diabetes mellitus or dyslipidemia, LMT or LAD as the culprit lesion, and renal deficiency were significantly associated with incidence of OHCA; (3) 30-day mortality was six times higher in the OHCA group than the non-OHCA group even after emergency revascularization; (4) renal deficiency, LMT or LAD as the culprit lesion, and high blood glucose level were independent predictors of 30-day mortality in the OHCA group (Fig. 3).

### Short-term prognosis of patients with AMI complicated by OHCA

Cardiac arrest is a fatal complication of AMI. Previous observational registries have reported that 5–8% of AMI patients presented with CA, and that short-term mortality was much higher in this group than in those without CA [7, 19], consistent with the findings of the present study. Approximately one-third of the present OHCA group died within 30 days of admission; however, we might have underestimated the prognostic impact of OHCA on AMI by excluding patients who were not indicated for emergency PCI due to their poor clinical condition. In addition, it is likely that many OHCA patients complicated with AMI could not be resuscitated successfully or transported to PCI-capable facilities. Successful resuscitation relies on a strong chain of survival, with the community, ambulance, and hospital working together. In the present study, 41 patients were in cardiac arrest on arrival, some of whom were resuscitated by defibrillation or advanced cardiac life support after arrival. In contrast, 33 patients in the OHCA group received percutaneous cardiopulmonary support (PCPS). PCPS was used aggressively in patients who could not be resuscitated successfully. Several studies have shown the benefit of extracorporeal cardiopulmonary resuscitation (CPR) in CA patients [20].

### Predictors of OHCA among the AMI population

Patients with OHCA were more likely to have LMT or LAD as the culprit lesions and accordingly large areas of infarction or ischemia, which are associated with poor outcome. In addition to large infarction or LMT culprit lesions, Kosugi et al. reported that chronic total occlusion was also associated with OHCA [21]. In contrast, multivessel coronary artery disease was not an independent factor for OHCA in the present study. Furthermore, patients in the present OHCA group were less likely to have coronary risk factors such as diabetes mellitus or dyslipidemia compared with the non-OHCA group. It is surprising that the absence of these comorbidities, which are well-known risk factors for cardiovascular disease, was associated with OHCA among AMI patients. Several previous studies have also reported that the classical coronary risk factors of hypertension, dyslipidemia, and diabetes mellitus are associated with a lower rate of CA among AMI patients [7, 8, 21]. One possible explanation is that these patients would have poor tolerance to acute ischemic events because of the lack of exposure to chronic ischemia [22] and would have a high risk of OHCA once MI had occurred. In addition, patients with coronary risk factors might be treated carefully by their physicians and some medications such as statins could improve clinical outcomes. In the present study, the rate of NSTEMI was higher in the OHCA group compared with the non-OHCA group. A recent study also reported a higher rate of NSTEMI in an OHCA group than in a non-OHCA group (24% vs. 22%) [21]. In the case of large areas of infarction or ischemia, ECG sometimes shows ST depression in several leads without ST elevation. In addition, we might have excluded OHCA patients with STEMI who could not transfer to our hospital or undergo emergency CAG because of their poor clinical condition. The present study included patients admitted during 2004–2017, and the need for emergency PCI has changed during this period. It is possible that the inclusion criterion has affected our results.

### Predictors of short-term mortality among patients with AMI complicated by OHCA

Consistent with the findings of a previous report [21], renal deficiency and LMT or LAD as the culprit lesion were associated with worse short-term mortality. Patients with renal deficiency have been reported to have a risk of CA before revascularization and to develop VF at the onset of MI [23, 24]. LMT or LAD as the culprit lesion is also a well-known risk factor of AMI mortality [25]. In particular, proximally located LAD as the culprit lesion had higher risk compared with non-proximally located LAD, which supports the finding that larger areas of infarction were associated with worse clinical outcome. It has also been reported that hyperglycemia causes worse mortality in AMI patients [26], which can be explained by the theory that the stress reaction in serious illness and in adverse glycometabolic effects such as vascular inflammation, a prothrombotic state, and higher free fatty acid concentrations is related to worse mortality of hyperglycemic patients with AMI [26, 27]. More intensive and careful treatments in the cardiac care unit after revascularization should be provided for patients with such predictors. However, there are no established treatment interventions for predictors such as early renal replacement therapy, complete revascularization, or active use of mechanical support and control of blood glucose to an appropriate level.

### Emergency PCI in AMI complicated by OHCA

As mentioned above, some of the patients with AMI complicated by OHCA were not indicated for emergency CAG or revascularization because of their poor clinical condition. Previous observational studies have reported an association between early treatment and better clinical outcomes [7, 28, 29], and therefore, emergency CAG and revascularization procedures are recommended in established guidelines for patients with STEMI [14, 30]. However, the role of urgent CAG in patients without ST-elevation is less clear. A recent meta-analysis has reported no significant differences in 30-day mortality, neurological status, or rate of PCI between OHCA patients without ST-elevation who were treated with early versus non-early CAG [31]. Thus, in patients without ST-elevation, it would be reasonable to perform immediate evaluation in the emergency department to exclude non-coronary causes and urgently perform echocardiography and blood tests.

### Limitations

This study has several limitations. First, as a single-center, observational study with a small patient cohort, unknown confounding factors could have affected the outcomes, regardless of analytical adjustments. A further large-scale prospective, multicenter study would be needed to confirm our findings. New information regarding treatments for OHCA patients could improve their outcomes. Second, it is possible that some patient data such as medical history and comorbidity might not have been collected, particularly in patients with OHCA. Third, as mentioned above, the prognostic impact of OHCA on AMI might be underestimated by excluding patients who could not undergo emergency PCI due to their poor clinical condition. In addition, it is possible that many patients could not be transferred to our hospital. Fourth, the registry included patients over a long period. The majority of patients were diagnosed using cardiac troponin; however, creatine kinase MB was used as a cardiac biomarker in some patients. Furthermore, the need for emergent PCI, especially for NSTEMI, might have changed over the long duration of the study period, and its use depends on the protocols of individual institutions and the decisions of doctors performing the procedure. It is possible that this inclusion criterion itself, which generates a non-homogeneous sample, might have affected the present results. Fifth, we used data from a registry that included only patients who had undergone PCI. Therefore, we missed data for AMI patients who had not undergone emergent PCI. Finally, we combined patients with STEMI and NSTEMI. Because the number of participants was relatively small, we were unable to divide them to perform a stratified analysis.

## Conclusions

Even though we enrolled only AMI patients who underwent emergency PCI, 30-day mortality was six times greater in patients presenting with OHCA compared with those without OHCA. Younger age, absence of diabetes mellitus or dyslipidemia, LMT or LAD as the culprit lesion, and renal deficiency were independent predictors of OHCA. In addition, among patients with OHCA, higher blood glucose level on admission, LMT or LAD as the culprit lesion, and renal deficiency were associated with worse clinical outcomes.

## Data Availability

The datasets used and/or analyzed during the current study are available from the corresponding author on reasonable request. All data generated or analyzed during this study are included in this published article.

## Abbreviations

AMI: Acute myocardial infarction
BMI: Body mass index
BP: Blood pressure
CA: Cardiac arrest
CAD: Coronary artery disease
CAG: Coronary angiography
CI: Confidence interval
eGFR: Estimated glomerular filtration rate
HDL-C: High-density lipoprotein cholesterol
LAD: Left anterior descending artery
LDL-C: Low-density lipoprotein cholesterol
LMT: Left main trunk
NSTEMI: Non-ST-elevation myocardial infarction
OHCA: Out-of-hospital cardiac arrest
OR: Odds ratio
PCI: Percutaneous coronary intervention
STEMI: ST-elevation myocardial infarction

## References

[1] Goldberg RJ, Currie K, White K, Brieger D, Steg PG, Goodman SG, Dabbous O, Fox KA, Gore JM (2004). Six-month outcomes in a multinational registry of patients hospitalized with an acute coronary syndrome (the Global Registry of Acute Coronary Events [GRACE]). Am J Cardiol.

[2] Daida H, Miyauchi K, Ogawa H, Yokoi H, Matsumoto M, Kitakaze M, Kimura T, Matsubara T, Ikari Y, Kimura K (2013). Management and 2-year long-term clinical outcome of acute coronary syndrome in Japan: prevention of atherothrombotic incidents following ischemic coronary attack (PACIFIC) registry. Circ J.

[3] Goldberg RJ, Gore JM, Alpert JS, Osganian V, de Groot J, Bade J, Chen Z, Frid D, Dalen JE (1991). Cardiogenic shock after acute myocardial infarction. Incidence and mortality from a community-wide perspective, 1975 to 1988. N Engl J Med.

[4] Killip T, Kimball JT (1967). Treatment of myocardial infarction in a coronary care unit. A 2 year experience with 250 patients. Am J Cardiol.

[5] Thiele H, Akin I, Sandri M, Fuernau G, de Waha S, Meyer-Saraei R, Nordbeck P, Geisler T, Landmesser U, Skurk C (2017). PCI strategies in patients with acute myocardial infarction and cardiogenic shock. N Engl J Med.

[6] Hochman JS, Sleeper LA, Webb JG, Sanborn TA, White HD, Talley JD, Buller CE, Jacobs AK, Slater JN, Col J (1999). Early revascularization in acute myocardial infarction complicated by cardiogenic shock. SHOCK Investigators. Should we emergently revascularize occluded coronaries for cardiogenic shock. N Engl J Med.

[7] Karam N, Bataille S, Marijon E, Tafflet M, Benamer H, Caussin C, Garot P, Juliard JM, Pires V, Boche T (2019). Incidence, mortality, and outcome-predictors of sudden cardiac arrest complicating myocardial infarction prior to hospital admission. Circ Cardiovasc Interv.

[8] Garot P, Lefevre T, Eltchaninoff H, Morice MC, Tamion F, Abry B, Lesault PF, Le Tarnec JY, Pouges C, Margenet A (2007). Six-month outcome of emergency percutaneous coronary intervention in resuscitated patients after cardiac arrest complicating ST-elevation myocardial infarction. Circulation.

[9] Takahashi M, Kondo Y, Senoo K, Fujimoto Y, Kobayashi Y (2018). Incidence and prognosis of cardiopulmonary arrest due to acute myocardial infarction in 85 consecutive patients. J Cardiol.

[10] Patel N, Patel NJ, Macon CJ, Thakkar B, Desai M, Rengifo-Moreno P, Alfonso CE, Myerburg RJ, Bhatt DL, Cohen MG (2016). Trends and outcomes of coronary angiography and percutaneous coronary intervention after out-of-hospital cardiac arrest associated with ventricular fibrillation or pulseless ventricular tachycardia. JAMA Cardiol.

[11] Spaulding CM, Joly LM, Rosenberg A, Monchi M, Weber SN, Dhainaut JF, Carli P (1997). Immediate coronary angiography in survivors of out-of-hospital cardiac arrest. N Engl J Med.

[12] Kern KB, Lotun K, Patel N, Mooney MR, Hollenbeck RD, McPherson JA, McMullan PW, Unger B, Hsu CH, Seder DB (2015). Outcomes of comatose cardiac arrest survivors with and without ST-segment elevation myocardial infarction: importance of coronary angiography. JACC Cardiovasc Interv.

[13] Thygesen K, Alpert JS, Jaffe AS, Chaitman BR, Bax JJ, Morrow DA, White HD (2018). Fourth universal definition of myocardial infarction (2018). J Am Coll Cardiol.

[14] Ibanez B, James S, Agewall S, Antunes MJ, Bucciarelli-Ducci C, Bueno H, Caforio ALP, Crea F, Goudevenos JA, Halvorsen S (2018). 2017 ESC Guidelines for the management of acute myocardial infarction in patients presenting with ST-segment elevation: the Task Force for the management of acute myocardial infarction in patients presenting with ST-segment elevation of the European Society of Cardiology (ESC). Eur Heart J.

[15] Mancia G, Fagard R, Narkiewicz K, Redon J, Zanchetti A, Böhm M, Christiaens T, Cifkova R, De Backer G, Dominiczak A (2013). 2013 ESH/ESC guidelines for the management of arterial hypertension: the Task Force for the Management of Arterial Hypertension of the European Society of Hypertension (ESH) and of the European Society of Cardiology (ESC). Eur Heart J.

[16] Teramoto T, Sasaki J, Ishibashi S, Birou S, Daida H, Dohi S, Egusa G, Hiro T, Hirobe K, Iida M (2013). Diagnostic criteria for dyslipidemia. J Atheroscler Thromb.

[17] International Expert Committee (2009). International Expert Committee report on the role of the A1C assay in the diagnosis of diabetes. Diabetes Care.

[18] Matsuo S, Imai E, Horio M, Yasuda Y, Tomita K, Nitta K, Yamagata K, Tomino Y, Yokoyama H, Hishida A (2009). Revised equations for estimated GFR from serum creatinine in Japan. Am J Kidney Dis.

[19] Kontos MC, Scirica BM, Chen AY, Thomas L, Anderson ML, Diercks DB, Jollis JG, Roe MT, NCDR (2015). Cardiac arrest and clinical characteristics, treatments and outcomes among patients hospitalized with ST-elevation myocardial infarction in contemporary practice: a report from the National Cardiovascular Data Registry. Am Heart J.

[20] Kelly EM, Pinto DS (2019). Invasive management of out of hospital cardiac arrest. Circ Cardiovasc Interv.

[21] Kosugi S, Shinouchi K, Ueda Y, Abe H, Sogabe T, Ishida K, Mishima T, Ozaki T, Takayasu K, Iida Y (2020). Clinical and angiographic features of patients with out-of-hospital cardiac arrest and acute myocardial infarction. J Am Coll Cardiol.

[22] Reiter R, Henry TD, Traverse JH (2013). Preinfarction angina reduces infarct size in ST-elevation myocardial infarction treated with percutaneous coronary intervention. Circ Cardiovasc Interv.

[23] Piccini JP, Berger JS, Brown DL (2008). Early sustained ventricular arrhythmias complicating acute myocardial infarction. Am J Med.

[24] Dalal D, de Jong JS, Tjong FV, Wang Y, Bruinsma N, Dekker LR, Wilde AA (2012). Mild-to-moderate kidney dysfunction and the risk of sudden cardiac death in the setting of acute myocardial infarction. Heart Rhythm.

[25] Velders MA, van Boven N, Boden H, van der Hoeven BL, Heestermans AA, Jukema JW, de Jonge E, Kuiper MA, van Boven AJ, Hofma SH (2013). Association between angiographic culprit lesion and out-of-hospital cardiac arrest in ST-elevation myocardial infarction patients. Resuscitation.

[26] Deedwania P, Kosiborod M, Barrett E, Ceriello A, Isley W, Mazzone T, Raskin P (2008). Hyperglycemia and acute coronary syndrome: a scientific statement from the American Heart Association Diabetes Committee of the Council on Nutrition, Physical Activity, and Metabolism. Anesthesiology.

[27] Capes SE, Hunt D, Malmberg K, Gerstein HC (2000). Stress hyperglycaemia and increased risk of death after myocardial infarction in patients with and without diabetes: a systematic overview. Lancet.

[28] Dumas F, Cariou A, Manzo-Silberman S, Grimaldi D, Vivien B, Rosencher J, Empana JP, Carli P, Mira JP, Jouven X (2010). Immediate percutaneous coronary intervention is associated with better survival after out-of-hospital cardiac arrest: insights from the PROCAT (Parisian Region Out of hospital Cardiac ArresT) registry. Circ Cardiovasc Interv.

[29] Reynolds JC, Callaway CW, El Khoudary SR, Moore CG, Alvarez RJ, Rittenberger JC (2009). Coronary angiography predicts improved outcome following cardiac arrest: propensity-adjusted analysis. J Intensive Care Med.

[30] Callaway CW, Donnino MW, Fink EL, Geocadin RG, Golan E, Kern KB, Leary M, Meurer WJ, Peberdy MA, Thompson TM (2015). Part 8: Post-Cardiac Arrest Care: 2015 American Heart Association guidelines update for cardiopulmonary resuscitation and emergency cardiovascular care. Circulation.

[31] Verma BR, Sharma V, Shekhar S, Kaur M, Khubber S, Bansal A, Singh J, Ahuja KR, Nazir S, Chetrit M (2020). Coronary angiography in patients with out-of-hospital cardiac arrest without ST-segment elevation: a systematic review and meta-analysis. JACC Cardiovasc Interv.

